# Diagnosis and management of venereal campylobacteriosis in beef cattle

**DOI:** 10.1186/s12917-014-0280-x

**Published:** 2014-11-27

**Authors:** Isabelle Truyers, Tim Luke, David Wilson, Neil Sargison

**Affiliations:** University of Edinburgh, Royal (Dick) School of Veterinary Studies, Easter Bush Veterinary Centre, Farm Animal Practice, Roslin, Midlothian EH25 9RG UK; West Gippsland Veterinary Centre, 1 Bona Vista Road, Warragul, Victoria 3820 Australia

**Keywords:** Beef cattle, Campylobacteriosis, Venereal, Bull, Reproduction

## Abstract

**Background:**

Bovine venereal campylobacteriosis is caused by *Campylobacter fetus* subsp. *venerealis* and its glycerine-tolerant variant *Campylobacter fetus* subsp. *venerealis* biovars *intermedius*. The disease can be economically important when present in cattle herds, causing poor reproductive performance, embryo mortality and abortion. Sensitive and specific diagnostic tests are required in the diagnosis of infection and to inform and monitor disease control. Current tests include bacterial culture and fluorescent antibody testing of preputial sheath washings and an enzyme-linked immunosorbent assay and an agglutination test on vaginal mucus, although the predictive values of these tests can be inadequate in field investigations.

Artificial insemination is often considered as a simple control method for bovine venereal campylobacteriosis, but is impractical for many beef suckler herds where breeding takes place at pasture. Commercial vaccines are unavailable in the UK, while the efficacy of autogenous vaccines using a bacterial isolate from infected animals on a specific farm is at best unproven. Hence, for some infected herds, the development of an alternative control strategy based on segregation of potentially infected and uninfected animals in combination with culling or treatment would be desirable. This approach requires meticulous records and herd health management.

**Case presentation:**

In this paper we highlight difficulties in diagnosing bovine venereal campylobacteriosis and demonstrate the benefits of good record keeping when investigating poor reproductive performance in a beef suckler herd and establishing a herd-specific approach to bio-containment of the infectious cause.

**Conclusions:**

Bovine venereal campylobacteriosis is an economically important disease that should be considered in investigations of suckler herd subfertility problems. Control of the disease based on segregation of potentially infected and uninfected animals in combination with extensive culling can be achieved without the use of artificial insemination or vaccination, but requires meticulous records and strict adherence to herd biosecurity practices.

## Background

The genus *Campylobacter* contains two important pathogens of animals affecting mainly the reproductive and gastrointestinal tracts [1]. Bovine venereal campylobacteriosis is associated with poor reproductive performance, early embryonic death and abortion in cattle. The causal agent of this sexually transmitted disease is *Campylobacter fetus* subsp. *venerealis* (Cfv) which has been isolated from the reproductive tract of cattle and internal organs of aborted foetuses [1-3]. *Campylobacter fetus* subsp. *fetus* (Cff) is transmitted orally and colonises the intestines of cattle and sheep, inducing enteritis and abortion mostly in sheep and sporadically in cattle [2,3]. Both subspecies can be distinguished based on their mechanism of transmission, clinical presentation and biochemical features, such as glycerine tolerance in Cff. However glycerine-tolerant variants of Cfv have also been described and designated as *Campylobacter fetus* subsp. *venerealis* biovars *intermedius* (Cfvi) [4,5].

Bovine venereal campylobacteriosis is transmitted mainly by natural service, but infection may also be spread during artificial insemination (AI) using semen from infected bulls or through contaminated equipment [6]. Direct transmission between female cattle is unlikely [7,8], but bull-to-bull spread of infection can occur during mounting behaviour when animals are co-housed [8]. In heifers and cows the bacteria spread to the uterus and oviducts resulting in endometritis and salpingitis. Pathology is most pronounced 8 to 13 weeks after infection and has generally resolved within 4 to 5 months. Infection does not affect conception but will typically result in early embryonic death and thus delayed return to oestrus. Abortions can occur at any time but are most commonly detected at 4 to 6 months of gestation. The disease is generally self-limiting in females. Most cows will recover and conceive within 3 to 6 months post-infection and immunity persists for several years [9,10], however some may remain infected for considerably longer [7]. In contrast, in bulls the infection is asymptomatic and neither lesions nor protective immunity develop. The bacteria can colonise the crypts of the preputial epithelium and as bulls age, the size and number of these crypts increase allowing persistence of infection, referred to as chronic carrier status, and making diagnosis and treatment more difficult [11,12,9].

The first steps in the investigation of a potential bovine venereal campylobacteriosis problem are to review the herd reproductive history, to conduct a biosecurity audit and to establish the presence or absence of associated clinical signs. Fluorescent antibody tests (FATs) are commonly used for antigen detection in preputial washings and have a reported sensitivity and specificity of 92.6% and 88.9% respectively [13]. An enzyme-linked immunosorbent assay (ELISA) is available to detect antigen-specific secretory IgA antibodies in the vaginal mucus following abortion due to Cfv. These antibodies are long-lasting but false reactions are possible because of antibody fluctuation in individual animals [14]. Vaginal mucus agglutination tests (VMATs) are also commonly used to detect antibodies in vaginal mucus washings with a sensitivity of about 50% [7]. Both the ELISA and VMATs are nevertheless useful as a herd screening method for *Campylobacter fetus* infection [10,14]. Preputial and vaginal washings intended for bacterial culture and identification must be collected according to standard bacteriological protocols and where transport to a laboratory could take more than 6 hours, transport enrichment media (TEM) should be used to enhance the *Campylobacter* spp. survival rate [7,15]. Molecular methods such as polymerase chain reaction (PCR) and sequence analysis can also be used for the diagnosis of bovine venereal campylobacteriosis [5,16,17].

There is anecdotal evidence of spontaneous recovery from bovine venereal campylobacteriosis occurring in young bulls. Successful local and systemic antibiotic therapy has been reported in bulls less than 3 years old, while culling of older bulls is usually recommended [12,9]. Streptomycin is the most extensively used antibiotic, although streptomycin resistant strains of *C. fetus* have been reported [18,19]. Treatment regimes include infusion into the preputial cavity, systemic therapy or a combination of both. Treatment of infected heifers and cows is not recommended because results are poor and most females develop protective immunity enabling them to resist re-infection [9,10].

Potentially infected breeding bulls are frequently culled following the confirmation of bovine venereal campylobacteriosis in beef suckler herds, accompanied by a temporary switch from natural mating to AI. *C. fetus* has been detected in vaginal washings from cows more than 1 year after infection [20], hence natural mating is generally not resumed until at least 2 gestation periods after the instigation of AI and ideally not until the last female naturally mated by a potentially infected bull has left the herd to reduce the risk of these animals infecting susceptible bulls at subsequent matings. Commercial bovine venereal camplyobacteriosis vaccines are not available in the UK, but autogenous vaccines can be produced under licence from the Veterinary Medicines Directorate [21]. These have been used prophylactically in controlling bovine venereal campylobacteriosis [22] but vaccinated animals can still mechanically transmit the disease [23].

## Case presentation

### Background information

In September 2012 high barren rates prompted an investigation into the cause of the subfertility in a beef suckler herd in south-east Scotland. The herd of predominantly spring calving cows, mostly commercial crossbreeds and some pedigree Angus, used natural service with Charolais, Simmental and Angus bulls. A small number of pedigree Angus cows were mated during the winter to calve in the following autumn. Store calves were finished off-site and the farm also sold breeding bulls.

During the summer of 2012, 169 breeding females split into 6 groups (A – F) had been mated to 8 different bulls. The mating period for both heifers and cows was 9 weeks and bulls had been rotated after 5 weeks. Pregnancy diagnosis (PD) using trans-rectal ultrasonography was carried out 32 days after the first bull was removed (1^st^ PD) and those females that were found non-pregnant at 1^st^ PD were rescanned 6 weeks after removal of the second bull (2^nd^ PD). In total 22% of heifers and cows were found barren, a figure noticeably higher than in previous years. The pregnancy and barren rates for the different groups that prompted the disease investigation are shown in Table 1.

**Table 1:** **Pregnancy and barren rates after a 9 week mating period during the summer of 2012**

**Group**	**Pregnant**	**Barren**
A	30/32 (94%)	2/32 (6%)
B	29/33 (88%)	4/33 (12%)
**C**	**12/31 (39%)**	**19/31 (61%)**
D	16/18 (89%)	2/18 (11%)
**E**	**23/32 (72%)**	**9/32 (28%)**
F	22/23 (96%)	1/23 (4%)
**Total**	**132/169 (78%)**	**37/169 (22%)**

Low pregnancy and high barren rates for group C, group E and total prompting further investigation in bold.

Such high barren rates in beef suckler herds in south-east Scotland are typically due to a bull fertility problem, and in this case Bull 3 was at first sight implicated in particular because he had been used in both Groups C and E where the highest barren rates were identified (Table 2). Nevertheless a stepwise investigation into the problem was formulated and agreed.

**Table 2:** **Bull use during the 2012 summer mating period for Groups A to F**

**Group**	**A**	**B**	**C**	**D**	**E**	**F**
1^st^ bull	Bull 1	Bull 2	Bull 3	Bull 4	Bull 5	Bull 6
2^nd^ bull	Bull 7	Bull 1	Bull 8	Bull 5	Bull 3	Bull 4

Bulls were rotated 5 weeks into the 9 week breeding season.

The farm boundaries were stock-proof and the herd was accredited free from bovine viral diarrhoea (BVD) and vaccinated against *Leptospira hardjo*^a^ and BVD^b^ as part of a veterinary herd health plan. Breeding cattle received their primary courses of both vaccines prior to the start of the mating season and boosters were given annually. All breeding cattle of 2 years of age and older were screened annually for Johne’s disease and control measures were implemented (the herd seroprevalence was less than 3%).

The herd had bred its own replacement females for a number of years until the owner decided to purchase 17 replacement heifers at market in April 2012. These females had been placed in quarantine on arrival and tested by the local veterinary diagnostic laboratory^c^ to identify persistent infection with BVD and presence of Johne’s disease antibodies. Two of the bought-in heifers were found to be persistently infected with BVD and culled. All bought-in heifers tested seronegative for antibodies against Johne’s disease.

During previous years the herd also acquired a number of breeding bulls, each of which had been young virgin animals of equal or higher health status.

### Initial investigation of poor reproductive performance

Data were collected for the body condition scores of all breeding cattle commencing in May 2012, before and at the start of the mating period.

Trans-rectal ultrasonography was used to examine the reproductive tracts of each of the non-pregnant females.

All 8 stock bulls had been submitted to a two-stage pre-breeding evaluation involving physical examination and examination of semen collected via electro-ejaculation using a Lane Pulsator IV Electronic Ejaculator^d^ [24], 6 weeks before the start of the mating season. As part of the investigation the breeding evaluation was repeated in October 2012 for Bull 3 and Bull 8, when a libido and mating assessment was also performed.

In October 2012, Bull 3, Bull 8 and 6 non-pregnant heifers from Group C (3 homebred and 3 purchased in April 2012) were presented for further investigation into the possibility of infectious infertility. Blood samples taken from these 8 animals were submitted for infectious bovine rhinotracheitis (IBR) serology. To assess whether bovine venereal campylobacteriosis was present in the herd, single preputial and vaginal washings were collected from the 2 bulls and 6 heifers, respectively using 30 to 50 ml of warmed saline and disposable sterile syringes, polythene tubing and sample bottles. The samples were labelled with the animals’ identities and submitted to the local Disease Surveillance Centre^c^ for *Campylobacter* culture within two hours of collection. At this point bovine venereal trichomonosis was not considered as a likely differential diagnosis since the disease had not been recognised in Britain for many years.

In depth analysis of farm records, in particular from those groups with poorest pregnancy results, was carried out. These records consisted of hand written documents kept up-to-date by the farm manager and provided information on the management tag numbers of all females allocated to groups A to F and the bulls that were used in these respective groups including the dates they were moved in and out. They also showed whether or not the females were found pregnant at 1^st^ or 2^nd^ PD which gave an indication of which bulls they had conceived to, if any.

### Results of initial investigation of poor reproductive performance

There was no evidence of vitamin, mineral or trace-element deficiencies and while the summer of 2012 was exceptionally wet and cold resulting in poaching and poor grass growth, the heifers were well grown and adult cattle were consistently in good target body condition score of 3/5 – 3.5/5 throughout the investigation.

The majority of the non-pregnant females’ ovaries showed active follicular waves and/or corpora lutea on trans-rectal ultrasonographic examination and there was no evidence of widespread anoestrus or endometritis.

The findings of all breeding soundness evaluations performed on the bulls in this herd, either as part of routine herd management procedures or as part of this investigation, were satisfactory.

Serological testing for IBR revealed that all bulls and homebred females were positive, whereas the bought-in replacements tested negative indicating that it was unlikely that the virus circulated in Group C prior to the poor reproductive performance problem arising.

No *Campylobacter* spp were detected on culture of prepuce and vaginal washings.

In group C all females that were pregnant had conceived to Bull 3 during the first 5 weeks of the mating season. None were found pregnant to Bull 8, a young homebred virgin sire. In Group E, with the exception of one cow, again all females that were found pregnant conceived during the first 5 weeks of the mating season (Table 3).

**Table 3:** **Pregnancy and barren rates for Groups C and E**

**Group**		**C**	**D**	**Total**
Pregnant	1^st^ PD	12 (39%)	22 (69%)	35 (56%)
	2^nd^ PD	0	1 (3%)	
Barren		19 (61%)	9 (28%)	28 (44%)
Total		31	32	63

By the end of October 2012 no plausible diagnosis had been made to explain the high barren rates in Groups C and E. It was speculated that the poor results could have been due to transient bull problems that had resolved by the time of investigation.

### Follow-up investigation

In January 2013 pregnancy results from a group of homebred virgin pedigree Angus heifers were again very low, whereas those of a group of Angus cows were satisfactory. This prompted further investigation into the possibility of infectious infertility. *Campylobacter* and *Trichomonas* sampling kits containing Weybridge TEM, freeze-dried antibiotic, sterile distilled water, phosphate buffered saline and filters^e^ were obtained. Preputial washing samples were collected from Bulls 3, 5 and 8 using the technique described in the leaflet accompanying the kits, labelled and dispatched with guaranteed next day delivery to the diagnostic laboratory^f^.

To assess whether the problem was limited to the groups with high barren rates, or more widespread in the herd, preputial washings from all 5 other stock bulls present on farm were collected twice with a 3 week interval and submitted for *Campylobacter* FAT and culture.

### Results of follow-up investigation

The length of the winter mating period was 6 weeks for both heifers and cows. In the heifer group, with only 1 out of 9 females pregnant, Bull 5 was initially used but he became lame only a few days into the breeding period and was replaced by Bull 3 until he recovered. In the cow group 22 out 24 females were pregnant.

The prepuce washings of Bulls 3, 5 and 8 all gave positive *C. fetus* FAT results and Cfvi was cultured from the prepuce washings of Bull 3, all 3 *Trichomonas foetus* cultures were negative and *T. foetus* was not detected using direct microscopic examination.

Prepuce washings from the other 5 stock bulls present on the farm gave consistently negative *C. fetus* FAT results. *Campylobacter upsaliensis* was isolated from one sample taken from Bull 2 but was not considered to be significant. These results provided important support for the conclusion that the disease problem was restricted to those groups of heifers and cows that had been mated by Bulls 3, 5 and 8 subsequent to the summer of 2012.

Additionally, analysis of farm records identified that the infection most likely originated in Group C and having been introduced with heifers purchased at market in April 2012. Since the farm policy was to rotate bulls mid breeding season, Bull 3 subsequently transmitted the disease to Group E. As the problem remained undiagnosed until January 2013, Cfvi subsequently spread to the small group of pedigree Angus heifers that were mated during the following winter.

### Disease management

The results of the disease investigation enabled the formulation of a novel disease management strategy:

1. All heifers and cows from groups C, E and the pedigree Angus heifers that were scanned barren were culled (amounting to 27 heifers and 9 cows culled).
2. Heifers and cows from groups C, E and the pedigree Angus heifers that maintained pregnancy to term were kept separate from the other breeding cattle in the herd and re-bred the following mating season, with homebred virgin bulls which were slaughtered at the end of mating.
3. Bulls 3 and 8 were culled.
4. Bull 5 was treated using a combination of local and systemic therapy for 3 consecutive days. A warmed penicillin and streptomycin solution^g^ was used for the sheath lavage. The suspension was inserted into the preputial orifice and massaged along the preputial cavity for one minute. This was repeated every 10 minutes for 1 hour. A streptomycin solution^h^ was administered via intramuscular injection at a dose rate of 10 mg active ingredient per kg bodyweight.
  The success of treatment was monitored using preputial washings into TEM and submission of samples to the diagnostic laboratory^f^ for *Campylobacter* spp culture and FAT. Preputial washings were collected at 30 and 33 days after the last day of treatment. A third washing was collected 30 days after the last previous test.
5. The success of treatment and management interventions was monitored by assessing pregnancy and barren rates following consecutive mating period(s).

### Results of disease management

Successful treatment of Bull 5 was demonstrated by consistently negative *Campylobacter* spp. culture and FAT results, obtained from preputial washings collected on 3 occasions post-treatment. Furthermore, Bull 5 was used to mate virgin heifers only for 9 weeks during the 2013 summer mating period and achieved a pregnancy rate of 93%. A total of 194 females were mated for 9 weeks during the summer of 2013 of which 177 (91%) were found pregnant and 17 (9%) barren. This is similar to pregnancy and barren rates achieved in the herd in the years preceding the disease outbreak described here.

## Discussion

This study shows that the introduction of Cfvi to a beef suckler herd with a group of purchased heifers resulted in 36 of 72 (50%) heifers and cows in 3 groups being barren before the diagnosis was confirmed and preventive management instigated, compared to a target barren rate for this system of 6% [25]. Profitability of a suckler herd is directly related to the number of calves reared per cow or heifer served annually, hence the economic consequences of such a high barren rate alone would have been significant, let alone the costs associated with the diagnosis and management of the problem, emphasising the importance of effective herd biosecurity. While endometritis and abortions are reported clinical features of bovine venereal campylobacteriosis [7,8,10], the abortion rate in this herd did not exceed the normal target of 2% and no signs of vaginal discharge were observed.

Bacterial culture of preputial and vaginal washings was unhelpful in the initial investigation described, and only confirmed the presence of Cfvi in one of three bulls with positive *C. fetus* FAT results during the follow-up investigation. Routine culturing procedures for Cfv appear to have poor sensitivity and consequently the disease could have been missed in the initial investigation. The problems associated with the diagnosis of bovine venereal campylobacteriosis based on the isolation of the organism are complex and include reduced viability of the organism under normal atmospheric conditions and rapid overgrowth of more vigorously multiplying contaminating organisms [15]. Furthermore, the sensitivity of bacterial culture may vary between different diagnostic laboratories [26]. There also exists considerable variation between bulls in their ability to harbour Cfv and the time they remain carriers and even persistently infected cattle can become negative on culture but later the organism can be isolated successfully again [14]. A recommendation has previously been made that at least 12 vaginal mucus washings from barren cows and prepuce washings from all bulls should be collected [21], although this also relates to limitations of the VMAT, which was not used in this case. From our experience we recommend that multiple available tests are used in parallel to increase the probability of a correct diagnosis and/or that tests are carried out in series to increase their sensitivity.

While there was evidence of seroconversion against IBR in this herd and the presence of this virus can manifest itself as an increased barren cow rate [27], it was not considered as a contributing factor to the subfertility since there was no evidence of virus circulation during the mating period or in the months thereafter. The identification of endemic IBR infection during this investigation was nevertheless pertinent, and a vaccination programme to control any future outbreak of the disease was implemented.

While bovine venereal campylobacteriosis can be transmitted through AI, in the European Union all licensed bulls used for AI purposes are tested and disease-free [28]. Culling bulls and whole herd AI [7,9,21] is therefore reported to be a simple, albeit often expensive and impractical approach to controlling the disease. However, this approach was considered to be undesirable in this case, not least because the herd depended on the sale of pedigree breeding sires. The use of an emergency vaccine, with unknown efficacy, was also deemed inappropriate as a long-term solution. Instead, a herd-specific disease control programme was developed, underpinned by the detailed investigation of the problem showing the disease to be restricted to just 3 groups of females and 3 bulls.

This novel approach to the control of bovine venereal campylobacteriosis in a beef suckler herd involved culling of heifers and cows that were scanned barren and total segregation of potentially infected and Cfvi-free animals. Barren females were culled in an attempt to reduce the prevalence of the disease and remove those that could be barren due to chronic damage to their reproductive tract. Additionally culling barren heifers and cows and replacing them with bulling heifers is considered more economical than keeping unproductive females until the next mating season. Two of the infected bulls were culled, while a particularly valuable bull was treated and then tested to demonstrate elimination of Cfvi from the prepuce. In this case, the approach proved to be more practical and considerably less expensive than the alternative of adopting an indefinite whole herd AI programme. The success of this approach required meticulous records and strict adherence to herd biosecurity practices.

In theory the purchase of virgin heifers and young bulls should offer little or no risk of introducing bovine venereal campylobacteriosis. Bulls 3, 5 and 8 were all homebred and either virgin or had achieved good pregnancy results during previous mating seasons. However, investigation of the ages of some of the purchased heifers in this case showed that they may have been old enough to have been mated and found barren prior to being sold. While this is unproven, it nevertheless highlights the potential risks and responsibilities for both vendors and purchasers of breeding heifers. Whilst 2 of the bought-in heifers were found to be persistently infected with BVD, the strict biosecurity measures adopted in this herd, including isolation and testing of introduced animals, were effective at avoiding the major financial losses that could have resulted from introducing this disease. From January 2014 Scottish legislation specifies that an animal identified positive for BVD virus on the most recent test will be assumed persistently infected and is not allowed to move other than directly to slaughter.

## Conclusions

This investigation clearly shows the value of accurate record-keeping on farm including the length of the breeding season, bull-to-cow details and pregnancy rates, in addition to information from bull breeding soundness evaluations, bull power, herd health and vaccination status, biosecurity policies and nutritional management. It demonstrates that record-keeping can be particularly important when investigating poor reproductive performance and in the bio-containment of infectious disease.

## Endnotes

^a^Leptavoid-H, MSD Animal Health, UK.

^b^Bovidec, Novartis, UK.

^c^SAC Consulting, Veterinary Services, Allan Watt Building, Bush Estate, Penicuik, EH26 0QE, UK.

^d^Lane Manufacturing, Colorado, USA.

^e^Campyculture RAI 0800.

^f^Animal Health and Veterinary Laboratories Agency, Starcross, UK.

^g^Pen & Strep Suspension for Injection, 200 mg/ml Procain Penicillin, 250 mg/ml Dihydrostreptomycin Sulphate in aqueous suspension, Norbrook, UK.

^h^Devomycin D Solution for Injection, 150 mg/ml Streptomycin Sulphate, 150 mg/ml Dihydrostreptomycin Sulphate in aqueous suspension, Norbrook, UK.

## Abbreviations

Cfv: *Campylobacter fetus* subsp. *venerealis*
Cff: *Campylobacter fetus* subsp. *fetus*
Cfvi: *Campylobacter fetus* subsp. *venerealis* biovars *intermedius*
AI: Artificial insemination
FAT: Fluorescent antibody test
ELISA: Enzyme-linked immunosorbent assay
VMAT: Vaginal mucus agglutination test
TEM: Transport enrichment medium
PCR: Polymerase chain reaction
PD: Pregnancy diagnosis
BVD: Bovine viral diarrhoea
IBR: Infectious bovine rhinotracheitis
